# Early symptoms of autism spectrum disorders and association with Brazilian children's development and behavior

**DOI:** 10.1590/2317-1782/e20240306en

**Published:** 2025-07-07

**Authors:** Rafaela Silva Moreira, Marina Aguiar Pires Guimarães, Lívia de Castro Magalhães, Janaina Matos Moreira, Claudia Regina Lindgren Alves

**Affiliations:** 1 Departamento de Ciências da Saúde, Universidade Federal de Santa Catarina, Araranguá (SC), Brasil.; 2 Baby Gym, Vitória da Conquista (BA), Brasil.; 3 Departamento de Terapia Ocupacional, Escola de Educação Física, Fisioterapia e Terapia Ocupacional, Universidade Federal de Minas Gerais – UFMG - Belo Horizonte (MG), Brasil.; 4 Departamento de Pediatria, Faculdade de Medicina, Universidade Federal de Minas Gerais – UFMG - Belo Horizonte (MG), Brasil.

**Keywords:** Autism Spectrum Disorder, Infants, Child Development, Behavior, Screening

## Abstract

**Purpose:**

to examine the association between the early autism spectrum disorder (ASD) signs and developmental and behavioral performance of Brazilian children aged 18 to 34 months.

**Methods:**

A cross-sectional study with 221 children recruited at public healthcare and early education services. Early symptoms of ASD were screened using the Parental Observations of Social Interaction (POSI). Children's development and behavior were examined using the Ages and Stages Questionnaires (ASQ-3) and Survey of Well-being of Young Children (SWYC-BR). The results of children at risk for ASD were compared to the no-risk ones using Chi-square and t-test.

**Results:**

The overall frequency of children at risk for ASD (POSI-positive screening) was 33% and did not differ across children's age range and gender. Children at risk for ASD showed worse socio-emotional behaviors (p=0.004) and lower scores for overall development (p=0.0001), communication (p=0.0007), fine motor (p=0.04), and personal-social domains (p=0.01). Differences between groups varied according to children's age and across developmental/behavioral domains and were more evident in older children. Children aged 30 to 34 months presented significant differences in overall development (p=0.001), behavior (p=0.004), and the personal-social domains (p=0.03).

**Conclusions:**

The frequency of children at risk for ASD was higher than described in the literature. Also, the development and behavior of children at risk for ASD were significantly different from their peers and compatible with the presentation of ASD in young children. Our findings reinforce the need for systematic and holistic surveillance of child development during well-being visits to improve ASD early detection.

## INTRODUCTION

Autism spectrum disorder (ASD) is a neurodevelopmental disorder that is more common in males, manifests in early childhood, and persists throughout life^(1,2)^. It is characterized by persistent deficits in communication and social interaction, repetitive and restricted behavior and interest patterns, which may cause significant impairment in overall development, limitations in daily activities, and restrictions in social participation^(1,3)^.

Recent studies have registered an increase in the prevalence and incidence of ASD worldwide despite data heterogeneity regarding diagnostic criteria, children's age, ethnicity, and gender^(2,4,5)^. In 2014, it was estimated that 17 out of 1,000 North American four-year-old children had ASD. In 2020, the overall prevalence among children aged 4 was 21.5 per 1,000; for those aged 8, it was 27.6 per 1,000. ASD was 3.8 times as prevalent among boys as girls^(4)^. In low- and middle-income countries, including Brazil, data on the prevalence of ASD are scarce, especially among young children^(3)^. Population studies in Turkey^(6)^ and Vietnam^(7)^ estimated a prevalence of 0.8% in young children. In Brazil, the most well-known populational study identified 6.4% of children between 7 and 14 years old as suspected of having ASD, whereas only 0.3% were confirmed^(8)^.

Although specific ASD symptoms can be recognized around 18 months and ASD diagnosis confirmed by age 2, most children are still diagnosed at school age^(9-11)^. This long gap causes incredible stress to families^(8,12)^ and directly affects the child's prognosis^(13,14)^, as early suspicion and interventions are essential for better outcomes.

Earlier in life, ASD symptoms may be subtle, varied, and nonspecific^(3,9,12,15)^. The American Academy of Pediatrics recommends child developmental surveillance in all routine medical visits and universal screening for ASD, using specific instruments, at 18 and 24 months^(9,16)^. In the Brazilian National Health System (SUS), development and growth surveillance should be offered to all children at the primary health care level. The screening and diagnosis of ASD can be made by teams of the Family Health Strategy, but usually at risk children are referred to other levels of the System, where specialized professionals proceed with the investigation and early stimulation^(17-20)^. Incorporating universal ASD screening into clinical practice is challenging^(19,21)^.

Some tests are costly, require extra materials and training, and necessitate additional time for application and interpretation. These practical aspects frequently prevent the use of ASD screening tools by pediatricians and other health care professionals in their daily routine^(3,16,22)^. Furthermore, there is still a lack of tools specific for ASD screening in young children adapted to the Brazilian context and with evidence of validity^(12,17,19,20)^.

The Modified Checklist for Autism in Toddlers (M-CHAT)^(23)^ and the Parents' Observations on Social Interaction (POSI)^(22,24)^ are both simple and accessible questionnaires based on parents' reports^(9,16)^ and are compatible with universal ASD screening^(25)^. In North American children, both POSI and M-CHAT have demonstrated sensitivity above 75% in evaluating risk for autism^(10)^.

The M-CHAT is the most widely used instrument for ASD screening^(9,25)^. M-CHAT has 23 items, is available in several languages, including Brazilian Portuguese, and has been recently validated in Brazil^(26)^.

POSI is a brief seven-question questionnaire that compounds the Survey of Well-being of Young Children (SWYC), a developmental-behavioral screening instrument cross-culturally adapted to the Brazilian context^(24,27)^. It assesses social interaction, communication, and repetitive behaviors in children aged 18 to 34 months and takes less than five minutes to be answered and interpreted^(22)^. POSI has already been adapted to Brazilian Portuguese^(27)^, although cutoff points have yet to be established, and accuracy studies with Brazilian children have not yet been conducted.

Nevertheless, POSI is part of the Survey of Well-being of Young Children (SWYC), a comprehensive instrument designed to assess both child development and behavioral domains. SWYC also screens risk factors in the family context, helping to compose the puzzle of ASD diagnosis. Sheldrick *et al*. highlight the importance of reconciling the screening test results, the parents' and professionals' concerns, and the clinical observations when deciding when to refer or not a child suspected of having ASD^(28)^. Thus, POSI might also be a promising instrument to be used on a large scale in primary healthcare settings as part of an integral developmental evaluation, mainly in early ages.

Considering the challenges of suspecting and diagnosing ASD in early childhood and recognizing the need to evaluate tools that can enhance ASD screening in public healthcare settings, the present study aimed to examine the association between early ASD signs and developmental and behavioral performance of Brazilian children aged 18 to 34 months.

## METHODS

### Study design

This study is an observational cross-sectional study that was part of a larger cohort study that investigated the evidence of the validity of the SWYC Brazilian version questionnaires^(27)^. It was approved by the Research Ethics Committee of the Federal University of Minas Gerais (CAAE 0456.0.203.000-11).

### Sample

It was a convenience sample composed of 221 children aged 18 to 34 months, recruited from Basic Healthcare Units/Family Health Strategy (BHU/FHS) and at Early Childhood Education Centers (ECEC) in two Brazilian urban cities, as part of the sample of the larger study. Following the protocols of the larger study, parents were invited to participate when they visited the BHU/FHS for any reason or when they went to pick up their children at the ECEC. All children in the age range whose parents/caregivers answered both the POSI questionnaire and the other developmental and behavioral assessments during the recruitment period of the larger study were included in the present study. Children with a prior diagnosis of neuromotor, sensory, or cognitive disorders were excluded.

### Instruments

The researchers developed a structured questionnaire to assess the mothers' socio-demographic characteristics, such as education level and age at childbirth. The families' purchasing power was assessed using the Economic Classification Criterion of the Brazilian Market Studies Association (CECB), which classifies socioeconomic levels, ranging from the highest (Level A) to the lowest (Levels D-E)^(29)^.

Developmental assessments were performed using the Brazilian versions of the Survey of Well-being of Young Children (SWYC-BR)^(24,27)^ and the Ages and Stages Questionnaire (ASQ-3)^(30,31)^, both based on parents' reports. Beyond assessing classic developmental domains, such as motor, language, and cognitive milestones, the SWYC-BR also has specific screening questionnaires for socioemotional behavioral problems (PPSC) and difficulties in social interactions (POSI), providing a holistic approach to child development.

The SWYC-BR is a multidimensional instrument for screening children's developmental delays and behavioral problems from 2 to 65 months^(24)^. The Developmental Milestones questionnaire was used to assess the child's overall development. It has 10 questions per age range to assess motor, cognitive, and language skills. The maximum score is 20 points, with higher scores indicating better performance^(24)^. The Preschool Pediatric Symptom Checklist (PPSC) comprises 18 items related to the child's socio-emotional behaviors (externalizing and internalizing behaviors, attention problems, and parenting challenges). The maximum score is 36 points. The higher the child's scores, the higher the chances of behavioral problems and the need for further evaluation^(32)^.

The Parental Observation of Social Interaction (POSI) questionnaire, which is also part of the SWYC-BR, is based on the Diagnostic and Statistical Manual of Mental Disorders (DSM-IV and DSM-V) and the M-CHAT^(22,24)^. It includes items related to the child's social behavior, such as "Does your child look if you point to something across the room?" and "How does your child usually show you something he or she wants?". Parents answer how often the child exhibits such behaviors and which toys are preferred. The interpretation is based on the graphic design of the questionnaire; the maximum score is seven points (Figure S1 - Supplementary Material). Scores greater than or equal to three indicate that the child should be referred for specialized evaluation due to symptoms related to ASD^(24)^. In our sample, children with a positive screening were referred for diagnostic evaluation in the public healthcare system.

The Ages and Stages Questionnaire (ASQ-3) was used to assess the communication, problem-solving, personal-social, and fine and gross motor domains^(30)^. The ASQ-3 has been translated and validated into several languages, including Brazilian Portuguese^(31)^. The forms are age-specific and contain six items by domain. Each ASQ-3 item is scored on a three-point scale, and the maximum score per domain is 60 points. The higher the scores, the better the performance in that domain^(30)^.

### Procedures

All parents who consented to participate in the study signed an informed consent form. Parents were interviewed in a private room by the research team in the BHU/FHS or ECEC between June 2014 and December 2018. The questionnaires were answered in the following sequence: sample characterization, Developmental Milestones, PPSC, POSI, and ASQ-3. Interviews lasted about 25 minutes.

### Analysis plan

Categorical variables were described using frequency distributions. The frequency of children presenting symptoms related to ASD was calculated according to the children's age and gender. Children's characteristics and their mean scores in SWYC-BR and ASQ-3 questionnaires were compared to POSI results (negative and positive screening) using the Chi-square test and T-test, respectively. The significance level was set at 5%. The analyses were performed in the Epi Info 7.2.3.1 software.

## RESULTS

Table 1 presents the sample's characteristics. Mothers were the main respondents (81.4%). They were predominantly adults (85.4%) and had completed nine years of education or more (80.4%). The families had low or very low socioeconomic status (74.2%). Most children were boys (51.1%) and were born full-term (90.3%).

**Table 1:** Characteristics of the sample

Characteristics of mothers and families	Total (n=221)	
	N	%
Mother's education (years)*		
≤ 8	43	19.6
9-11	99	45.2
>11	77	35.2
Mother's age at child's birth (years)*		
≤ 19	32	14.6
>19	187	85.4
Socioeconomic classification (CECB)*		
A+B1+B2	54	25.8
C1+C2	133	63.7
D/E	22	10.53
First child*		
Yes	108	49.1
No	112	50.9
Characteristics of the children		
Gender		
Male	113	51.1
Female	108	48.9
Prematurity (weeks)		
<37	21	9.7
≥37	196	90.3
Age (months)		
18-23	46	20.8
24-29	88	39.8
30-34	87	39.4

CECB - Criteria for Economic Classification of the Brazilian Association of Market Research (ABEP)^(30)^

* data missing

Table 2 reports the frequency of children with positive screening in POSI by age group and gender. The overall frequency of children who should be referred to specialized evaluation for ASD was 33%. The frequency of positive POSI screening results did not differ across age groups and children's genders.

**Table 2:** Comparison of POSI results, according to children's age and gender

POSI	Total sample (N=221)					Male (N=113)					Female (N=108)				
	Negative screening		Positive screening		p^*^	Negative screening		Positive screening		p*	Negative screening		Positive screening		p^*^
Age in months	n	%	n	%		n	%	n	%		n	%	n	%	
18**–**23	32	69.6	14	30.4	0.93	14	60.8	9	39.1	0.80	18	78.2	5	21.7	0.58
24**–**29	57	64.8	31	35.2		27	62.7	16	37.2		30	66.6	15	33.3	
30**–**34	59	67.8	28	32.2		32	68.0	15	31.9		27	67.5	13	32.5	
Total	148	67.0	73	33.0	-	73	64.6	40	35.4	-	75	69.4	33	30.5	0.53**

* Chi-square;

** males *vs*. females

Table 3 presents children's developmental and behavioral performance according to POSI results. Compared to children with negative screening, children with positive screening for ASD showed lower scores in Developmental Milestones (p=0.0001), higher scores in PPSC (p=0.004), lower scores in communication (p=0.0007), fine motor (p=0.04), and personal-social (p=0.01) domains of the ASQ-3.

**Table 3:** Comparison of POSI results and mean scores on the SWYC-BR and ASQ-3 questionnaires

	POSI (N= 221)		
	Negative screening (n=148)	Positive screening (n=73)	p-value*
SWYC-BR questionnaires (Mean±SD)			
Developmental Milestones	15.6±3.5	13.4±4.3	**0.0001**
Preschool Pediatric Symptom Checklist (PPSC)	8.9±5.0	11.1±6.4	**0.004**
ASQ-3 domains (Mean±SD)			
Communication	51.3±10.6	45.4±14.1	**0.0007**
Fine motor	42.4±12.7	38.7±12.4	**0.04**
Gross motor	52.9±8.7	51.0±9.6	0.14
Personal-social	47.7±10.6	43.8±12.4	**0.01**
Problem solving	46.4±10.8	45.8±10.5	0.66

* T-test

Table 4 shows the analysis of the association between the POSI results and mean scores on the SWYC-BR and ASQ-3 domains by age range. For the younger children (18-23 months), in general, those with a positive screening in the POSI scored lower considering developmental milestones and ASQ-3 domains, although differences were significant only for the communication domain (p=0.01). This pattern was also observed in the other age groups, with significant differences in overall development (SWYC-BR) for children aged 24 months or older. Children aged 30-34 months with POSI-positive screening also scored significantly higher in PPSC (p=0.004) and significantly lower in the personal-social domain (p=0.03). Of note, the differences between children with positive and negative screening tended to be significant for those older than 24 months in the communication domain (p<0.10).

**Table 4:** Comparison of POSI results and mean scores on the SWYC-BR and ASQ-3 domains, according to children's age range

Instrument	18–23 months (n=46)			24–29 months (n=88)			30–34 months (n=87)		
	Negative screening (n=32)	Positive screening (n=14)	p-value*	Negative screening (n=57)	Positive screening (n=31)	p-value*	Negative screening (n=59)	Positive screening (n=28)	p-value*
SWYC-BR (Mean±SD)									
Developmental Milestones	15.7±3.3	14±3.8	0.13	15.7±3.7	13.8±5.2	**0.04**	15.3±3.7	12.6±3.4	**0.001**
Preschool Pediatric Symptom Checklist (PPSC)	8.5±4.5	10.8±5.6	0.14	8.8±5.4	9.7±6.2	0.48	9.1±5.1	12.9±6.9	**0.004**
ASQ-3 domains (Mean±SD)									
Communication	50.2±12.5	38.9±16.2	**0.01**	51.1±10.8	45.8±15.2	0.06	52.0±9.5	48.2±10.8	0.09
Fine motor	45.3±8.7	41.8±12.2	0.27	43.9±13.6	40.2±12.5	0.21	39.5±13.2	35.5±12.0	0.18
Gross motor	53.4±9.1	54.6±6.9	0.66	53.0±9.6	50.0±11.0	0.19	52.6±7.7	50.4±9.1	0.23
Personal-social	47.3±9.2	42.5±11.2	0.13	45.8±11.3	44.2±12.0	0.53	49.8±10.5	43.9±13.6	**0.03**
Problem solving	46.9±9.2	46.0±5.9	0.77	44.7±11.7	46.1±11.5	0.59	47.8±10.8	45.2±11.3	0.30

* T-test

## DISCUSSION

The present study analyzed the association between POSI results and child development and socio-emotional behavior. Early symptoms of risk for ASD were associated with children's performance in the developmental and behavioral questionnaires. We found that approximately one-third of the children were considered at-risk for ASD and would need a specialized reassessment. In general, the children identified as at-risk for ASD had lower scores on developmental scales and higher scores on behavioral assessment than those with a negative screening.

In the present study, the frequency of risk symptoms for ASD in children aged 18 to 34 months was higher than reported in other studies conducted in Brazil^(33,34)^. Considering only studies with children under three years old that applied M-CHAT, the suspicion of ASD ranged from 10.8%^(7,35)^ to 15.3%^(36)^, and the diagnostic confirmation rate ranged from 0.08%^(36)^ to 0.8%^(6,7)^. Important methodological differences, such as sample size, the nature of the tests, settings, children's age, and clinical profile, may explain the divergences across the studies. Moreover, the POSI is a pre-screening test, so a high number of false-positive cases can be expected. The sensitivity of POSI in North American children was very high, but its specificity was low^(10,22)^. In the original POSI validation study, the prevalence of positive screening was 12%, and the diagnosis of ASD was confirmed in 2.5% of at-risk children. However, around half of the children who screened positive on the POSI but ASD was not confirmed had developmental delays and other diagnoses, indicating that they could benefit from early intervention^(22)^. It highlights the importance of considering other signs and symptoms that may raise suspicions of ASD to increase the specificity of the POSI results.

Aligned with the literature^(9,11)^, our results showed that the developmental and behavioral performance of children considered at risk for ASD differed from those not at risk. The most significant differences were noted in domains compatible with early ASD manifestations, such as socioemotional behavior, communication, and social-personal skills^(1,9,35)^, meaning that other developmental delays and behavioral problems frequently accompanied the suspicious of ASD. ASD diagnosis is challenging in young children as signs and symptoms can be unspecific and subtle, so parents and professionals should value each other's concerns about the child's development and behaviors^(3,9,12,15)^. It will enable a quick response to children's needs, which will not necessarily be an ASD diagnosis. Therefore, the POSI results observed in the present study should be understood as just one necessary piece of information to help professionals decide whether to proceed with the investigation for ASD.

In the present study, we observed an association between the outcomes of children at risk for ASD and their global development, using both the SWYC-BR and the ASQ-3. We found that the mean scores in the communication and social-personal domains of the ASQ-3 were lower in children at risk of ASD. Similar results were observed in a study that examined the ability of the ASQ-3 to predict the risk of ASD in 2848 North American children aged 16 to 30 months^(35)^. About 70% of the children with a positive ASD screening (M-CHAT) scored lower than expected in at least one of the ASQ-3 domains. Among the 21 children with a confirmed diagnosis of ASD, 95% presented impairment in communication, 71% in social-personal, and 66% in fine motor skills. The authors reinforce the importance of longitudinal monitoring of overall development to detect children at risk of ASD^(35)^.

Considering the results according to age groups, we found that communication delays were the first and most affected domain. They were present in the youngest children at risk for ASD and tend to be present in the oldest children, too. Similarly, Li et al.^(37)^ found that autistic traits at age 3 were predicted by expressive language at 14 months and receptive language at 24 months. In children older than 24 months, differences in overall development were more evident, reflecting a global delay in cognitive, language, and motor skills. Probably, the youngest children do not yet show signs of developmental delays that indicate a risk of ASD, the signs becoming more visible to parents from age 2. Sacrey et al.^(38)^, in a prospective study of children aged 6 to 36 months at risk of ASD, found that parental reports of concerns about sensory, motor, and play aspects in the first year of life and communication and social skills after the second year of life were predictive of ASD.

Children between 30 and 34 months at risk of ASD showed fewer personal and social skills, more internalizing and externalizing symptoms, and attention difficulties than their peers. These findings indicate that behavioral challenges were better observed from the age of 30 months and, despite not being part of the ASD diagnostic criteria, can be predictors of other neurodevelopmental disorders. Behavioral problems are one of the biggest concerns of parents of children at risk of ASD because they directly affect families' social lives and children's adaptation to a variety of environments^(1,9)^. These findings corroborate the need for a holistic approach to child development, looking at socioemotional behaviors and parents' concerns as well as the classic developmental milestones. The early diagnosis of autism is a difficult but extremely important task. Therefore, it is necessary to gather all the symptoms and relevant information in order to help children access early intervention, even if they have not yet been diagnosed.

This study has strengths but also some limitations. The sample was relatively large compared to previous Brazilian studies. Participants were recruited from two cities with different contexts and included children who presented no previous known risks for ASD. However, it was a convenience sample, so the results should not be generalized. We used internationally recognized instruments to screen for developmental delays and behavioral problems based on parents' reports, which may have overestimated the frequency of children at risk for ASD. Moreover, most families had low and very low socioeconomic status, which may indicate that children were exposed to a variety of environmental risks for developmental delays related to poverty. Conversely, the differences observed in children's performance with and without risk for ASD indicate the consistency of the results.

It is worth noting that, to our knowledge, this is the first study using the POSI to screen for the risk of ASD in Brazil. Although many measurement properties of the SWYC-BR have already been established, the lack of normative data on POSI for Brazilian children requires caution when interpreting its results. On the other hand, our findings bring some evidence of the POSI construct validity, as we showed the coherence between POSI results and the other screenings. The availability of the Brazilian version of the SWYC represents an advance in assessing child development with a holistic approach, and its use in public health care settings may contribute to the early identification of children at risk of ASD as well as other developmental and behavioral problems.

## CONCLUSIONS

The frequency of children at risk for ASD was higher than described in the literature. Also, the development and behavior of children at risk for ASD were significantly different from their peers and compatible with the presentation of ASD in young children. Our findings reinforce the importance of standardized measures that provide a broader perspective on development, behavior, and family context, including early childhood ASD screening tools like the POSI. Employing instruments that assess multiple domains can benefit primary healthcare providers by enhancing the amount of information gathered and reducing the time required to administer multiple tools. Early identification of children at risk for ASD through developmental monitoring may present a cost-effective alternative for society and holds promise for facilitating timely interventions, thus maximizing skill development during this critical period of development.

## Supplementary Material

Supplementary material accompanies this paper.

Figure S1. POSI questionnaire

This material is available as part of the online article from https://doi.org/10.1590/2317-1782/e20240306en
