# Supplementary material for: Early symptoms of autism spectrum disorders and association with Brazilian children's development and behavior
Source: Codas. 2025 Jul 7;37(4):e20240306. doi: 10.1590/2317-1782/e20240306en (PMC12259063; doi:10.1590/2317-1782/e20240306en)
Supplement: Figure S1. [file codas-37-4-e20240306-suppl01.pdf]

Supplementary material

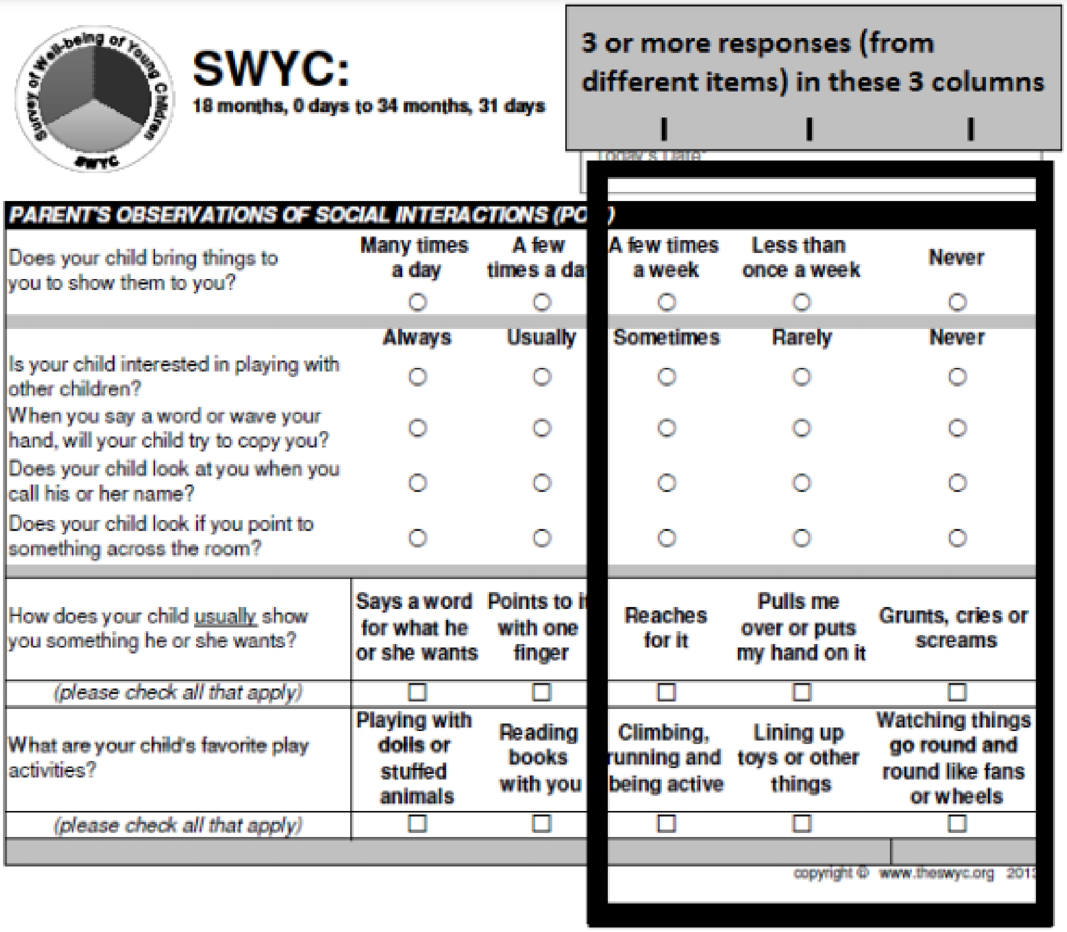

3 or more responses (from different items) in these 3 columns

SWYC:

18 months, 0 days to 34 months, 31 days

Today's Date:

PARENT'S OBSERVATIONS OF SOCIAL INTERACTIONS (PO[illegible])

Does your child bring things to you to show them to you?	Many times a day ○	A few times a da[illegible] ○	A few times a week ○	Less than once a week ○	Never ○
	Always	Usually	Sometimes	Rarely	Never
Is your child interested in playing with other children?	○	○	○	○	○
When you say a word or wave your hand, will your child try to copy you?	○	○	○	○	○
Does your child look at you when you call his or her name?	○	○	○	○	○
Does your child look if you point to something across the room?	○	○	○	○	○
How does your child usually show you something he or she wants?	Says a word for what he or she wants	Points to i[illegible] with one finger	Reaches for it	Pulls me over or puts my hand on it	Grunts, cries or screams
(please check all that apply)	☐	☐	☐	☐	☐
What are your child's favorite play activities?	Playing with dolls or stuffed animals	Reading books with you	Climbing, [illegible]unning and being active	Lining up toys or other things	Watching things go round and round like fans or wheels
(please check all that apply)	☐	☐	☐	☐	☐

copyright © www.theswyc.org 201[illegible]

Figure S1 – POSI questionnaire

Interpretation: A result of three or more points in the last three columns indicates that a child is "at-risk" and needs further evaluation or investigation. (Perrin et al., 2016)
